# ^1^H-enhanced ^103^Rh NMR spectroscopy and relaxometry of ^103^Rh(acac)_3_ in solution

**DOI:** 10.5194/mr-5-121-2024

**Published:** 2024-08-30

**Authors:** Harry Harbor-Collins, Mohamed Sabba, Markus Leutzsch, Malcolm H. Levitt

**Affiliations:** 1 School of Chemistry, University of Southampton, SO17 1BJ, Southampton, UK; 2 Service Department NMR Spectroscopy, Max-Planck-Institut für Kohlenforschung, Kaiser-Wilhelm-Platz 1, 45470 Mülheim an der Ruhr, Germany

## Abstract

Recently developed polarisation transfer techniques are applied to the ${}^{103}\mathrm{Rh}$ nuclear magnetic resonance (NMR) of the ${}^{103}\mathrm{Rh}(\mathrm{acac}{)}_{3}$ coordination complex in solution. Four-bond ${}^{1}H$ – ${}^{103}\mathrm{Rh}$ J couplings of around 0.39 Hz are exploited to enhance the ${}^{103}\mathrm{Rh}$ NMR signal and to estimate the ${}^{103}\mathrm{Rh}$ $T_{1}$ and $T_{2}$ relaxation times as a function of field and temperature. The ${}^{103}\mathrm{Rh}$ longitudinal $T_{1}$ relaxation in ${}^{103}\mathrm{Rh}(\mathrm{acac}{)}_{3}$ is shown to be dominated by the spin–rotation mechanism, with an additional field-dependent contribution from the ${}^{103}\mathrm{Rh}$ chemical shift anisotropy.

## 1 Introduction

Although rhodium is one of the few chemical elements with a 100 % abundant spin- 1/2 isotope, the routine nuclear magnetic resonance (NMR) of ${}^{103}\mathrm{Rh}$ has been inhibited by its very small gyromagnetic ratio, which is negative and ∼ 31.59 times less than that of ${}^{1}H$ ([Bibr bib1.bibx35]).

While indirectly detected ${}^{103}\mathrm{Rh}$ NMR has had an appreciable history ([Bibr bib1.bibx15]), advances in instrumentation and methodology have allowed rapid observation of ${}^{103}\mathrm{Rh}$ NMR parameters on standard commercial NMR spectrometers, leading to a recent renaissance of the field ([Bibr bib1.bibx11]).

The rhodium(III) acetylacetonate ( ${}^{103}\mathrm{Rh}(\mathrm{acac}{)}_{3}$ ) complex (see Fig. [Fig Ch1.F1]) currently serves as the International Union of Pure and Applied Chemistry (IUPAC) ${}^{103}\mathrm{Rh}$ NMR chemical-shift reference ([Bibr bib1.bibx9]). To the wider scientific community, ${}^{103}\mathrm{Rh}(\mathrm{acac}{)}_{3}$ is better known for its role in the production of thin rhodium films and nanocrystals for use in catalysis ([Bibr bib1.bibx49]).

The early studies of nuclear spin relaxation in ${}^{103}\mathrm{Rh}(\mathrm{acac}{)}_{3}$ were greatly limited by the poor ${}^{103}\mathrm{Rh}$ signal strength, and they provided somewhat conflicting conclusions for the ${}^{103}\mathrm{Rh}$ relaxation mechanisms ([Bibr bib1.bibx22]). Recently, a two-bond ${}^{13}C$ – ${}^{103}\mathrm{Rh}$ coupling of 1.1 Hz was observed in ${}^{103}\mathrm{Rh}(\mathrm{acac}{)}_{3}$ and was exploited for triple-resonance experiments ([Bibr bib1.bibx8]).

We now report the observation of a four-bond ${}^{1}H$ – ${}^{103}\mathrm{Rh}$ J coupling of ${|}^{4}J_{\text{HRh}}|$ ≃ 0.39 Hz between the central ${}^{103}\mathrm{Rh}$ nucleus and each of the three methine ${}^{1}H$ nuclei in ${}^{103}\mathrm{Rh}(\mathrm{acac}{)}_{3}$ (see Fig. [Fig Ch1.F1]). These small couplings are exploited for the ${}^{1}H$ -enhanced ${}^{103}\mathrm{Rh}$ NMR spectroscopy of the ${}^{103}\mathrm{Rh}(\mathrm{acac}{)}_{3}$ complex. ${}^{103}\mathrm{Rh}$ spin–lattice $T_{1}$ and spin–spin $T_{2}$ relaxation time constants are measured over a range of magnetic fields and temperatures. The ${}^{103}\mathrm{Rh}$ $T_{1}$ relaxation is found to be dominated by spin–rotation, with an additional contribution from the chemical shift anisotropy (CSA), which is significant at high fields.

**Figure 1 Ch1.F1:**
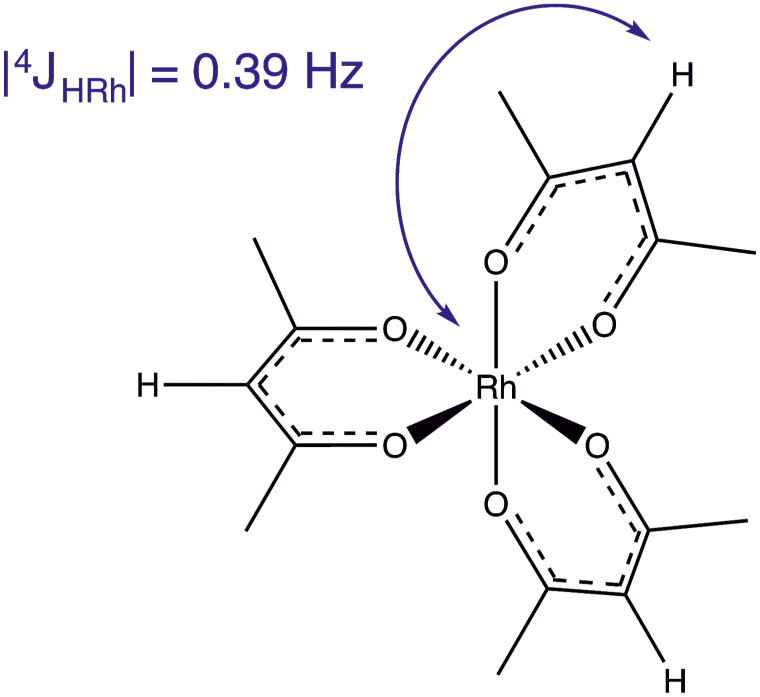
The molecular structure of rhodium(III) acetylacetonate, ${}^{103}\mathrm{Rh}(\mathrm{acac}{)}_{3}$ , which has point group symmetry $D_{3}$ . This work exploits the long-range ${}^{4}J_{\text{HRh}}$ scalar couplings for polarisation transfer between the ${}^{103}\mathrm{Rh}$ and methine ${}^{1}H$ spins.

## 2 Experimental

Experiments were performed on a saturated ( ∼ 140 mM ) solution of rhodium(III) acetylacetonate ( ${}^{103}\mathrm{Rh}(\mathrm{acac}{)}_{3}$ ) dissolved in 350 µL ${\mathrm{CDCl}}_{3}$ . ${}^{103}\mathrm{Rh}(\mathrm{acac}{)}_{3}$ was purchased from Sigma-Aldrich and used as received. ${}^{103}\mathrm{Rh}(\mathrm{acac}{)}_{3}$ is a bright yellow powder, which is dissolved in ${\mathrm{CDCl}}_{3}$ to form a solution with a deep golden colour.

The radio frequency channels were additionally isolated by installing a bandpass (K&L Microwave) and low-pass (Chemagnetics 30 MHz ) filter at the preamplifier outputs of the ${}^{1}H$ and ${}^{103}\mathrm{Rh}$ channels, respectively. Pulse powers on the ${}^{1}H$ and ${}^{103}\mathrm{Rh}$ channels were calibrated to give a matched nutation frequency of 2 π × 4000 Hz , corresponding to a 90° pulse length of 62.5 µs . Field-cycling experiments were performed using a motorised fast-shuttling system ([Bibr bib1.bibx50]). The shuttling time was kept constant at 2 s , in both directions.

## 3 Results

### 3.1 ${}^{1}H$ spectrum

The ${}^{1}H$ spectrum for ${}^{103}\mathrm{Rh}(\mathrm{acac}{)}_{3}$ in ${\mathrm{CDCl}}_{3}$ (shown in Fig. [Fig Ch1.F2]a) features two resonances: a singlet at 2.170 ppm , corresponding to the six methyl protons on each acac ligand, and a broad, weak doublet centred at 5.511 ppm , corresponding to the acac methine protons. An expanded region showing just the methine resonance is presented in Fig. [Fig Ch1.F2]b. The four-bond ${}^{103}\mathrm{Rh}$ – ${}^{1}H$ spin–spin coupling is estimated to be ${|}^{4}J_{\text{HRh}}|$ = 0.39 ± 0.01 Hz .

**Figure 2 Ch1.F2:**
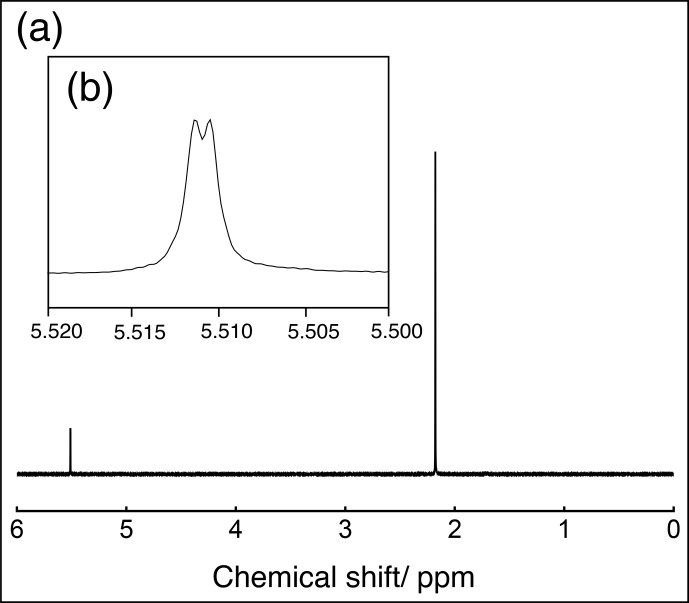
**(a)** ${}^{1}H$ spectrum of a ∼ 140 mM solution of ${}^{103}\mathrm{Rh}(\mathrm{acac}{)}_{3}$ in ${\mathrm{CDCl}}_{3}$ , acquired at 9.4 T and 298 K , in a single transient. Lorentzian line broadening (1 Hz ) was applied. **(b)** Expanded view of the methine ${}^{1}H$ resonance. Negative Lorentzian line broadening ( - 0.2 Hz ) was applied to enhance the resolution.

### 3.2 ${}^{103}\mathrm{Rh}$ spectra

#### 3.2.1 Direct ${}^{103}\mathrm{Rh}$ excitation

The ${}^{1}H$ -decoupled ${}^{103}\mathrm{Rh}$ spectrum of the ${}^{103}\mathrm{Rh}(\mathrm{acac}{)}_{3}$ solution, acquired with single-pulse excitation of ${}^{103}\mathrm{Rh}$ transverse magnetisation, is shown in Fig. [Fig Ch1.F3]a and displays a single peak with the ${}^{103}\mathrm{Rh}$ chemical shift of 8337.6 ppm . The signal-to-noise ratio is quite poor, even after 12 h of data acquisition.

**Figure 3 Ch1.F3:**
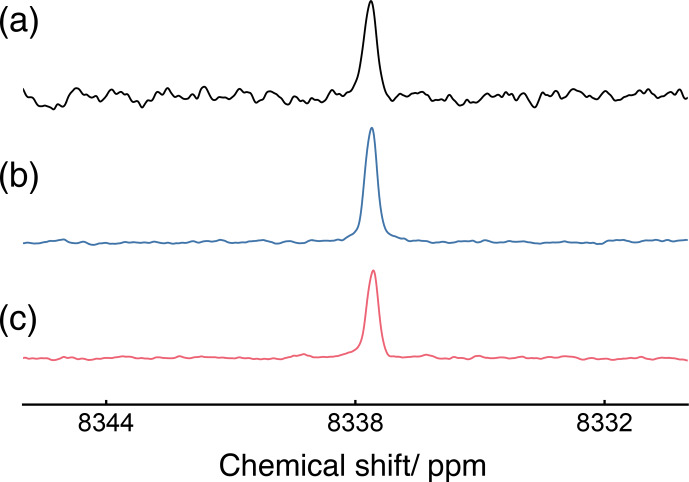
${}^{1}H$ -decoupled ${}^{103}\mathrm{Rh}$ NMR spectra of a ∼ 140 mM solution of ${}^{103}\mathrm{Rh}(\mathrm{acac}{)}_{3}$ in ${\mathrm{CDCl}}_{3}$ , in a field of 9.4 T and at a temperature of 295 K . Lorentzian line broadening (1 Hz ) was applied to all spectra. ${}^{103}\mathrm{Rh}$ chemical shifts are referenced to the absolute frequency ( $\Xi ({}^{103}\mathrm{Rh})$ = 3.16 %). In all spectra, ${}^{1}H$ decoupling was achieved using continuous wave decoupling with 0.05 W of power, corresponding to a nutation frequency of 1 kHz . **(a)** ${}^{103}\mathrm{Rh}$ Hdec spectrum, acquired using 300 transients, each using a single ${}^{103}\mathrm{Rh}$ 90° pulse. The waiting interval between transients was 150 s . The total experimental duration was ∼ 12 h . **(b)** ${}^{103}\mathrm{Rh}$ Hdec spectrum, acquired using 16 transients and the pulse sequence shown in Fig. [Fig Ch1.F4], with n=11 repetitions of the DualPol (dual-channel PulsePol) sequence. The waiting interval between transients was 18 s . The total experimental duration was 5 min . **(c)** ${}^{103}\mathrm{Rh}$ Hdec spectrum, acquired using 16 transients and an optimised refocused-INEPT (Insensitive Nucleus Enhancement by Polarisation Transfer) sequence. The waiting interval between transients was 18 s . The total experimental duration was 5 min .

#### 3.2.2 ${}^{1}H$ – ${}^{103}\mathrm{Rh}$ polarisation transfer by DualPol

Polarization transfer from the ${}^{1}H$ nuclei to the ${}^{103}\mathrm{Rh}$ nuclei was performed using the previously described DualPol pulse sequence incorporating acoustic-ringing suppression ([Bibr bib1.bibx25]), as shown in Fig. [Fig Ch1.F4].

**Figure 4 Ch1.F4:**
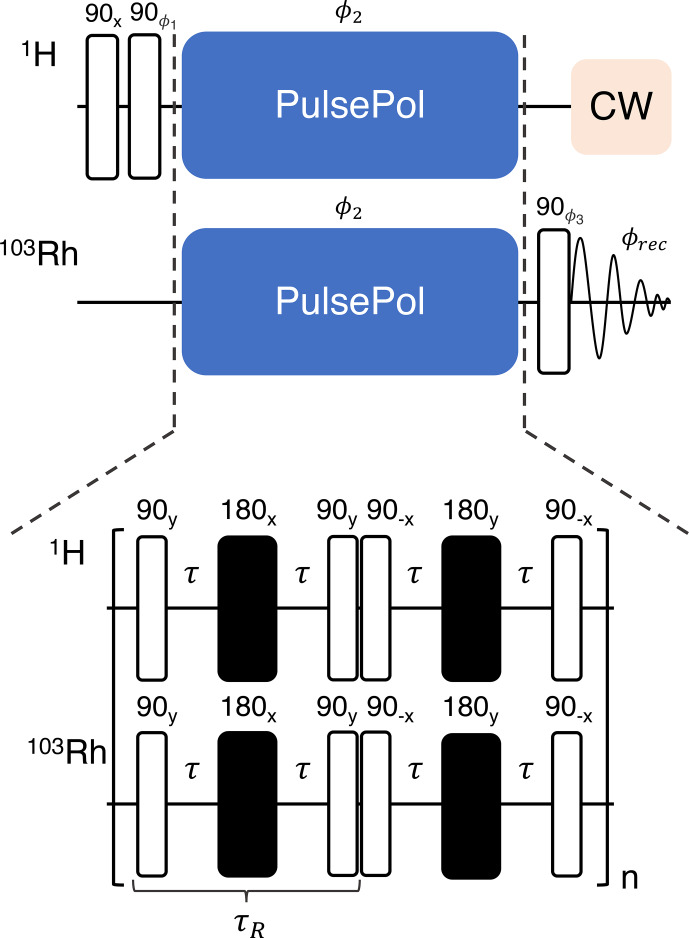
Pulse sequence for the acquisition of ${}^{1}H$ -enhanced ${}^{103}\mathrm{Rh}$ spectra; an expanded view of the DualPol pulse sequence module is shown at the bottom. The black rectangles indicate symmetrised BB1 composite 180° pulses ([Bibr bib1.bibx48]) and τ indicates an interpulse delay. Phase cycles are given by the following: $\phi_{1}=[-x,x,-x,x]$ , $\phi_{2}=[x,x,-x,-x]$ , $\phi_{3}=[x,x,x,x,y,y,y,y,-x,-x,-x,-x,-y,-y,-y,-y]$ and the receiver $\phi_{\text{rec}}=[x,-x,x,-x,y,-y,y,-y,-x,x,-x,x,-y,y$ , -y,y] .

The DualPol sequence consists of two synchronised PulsePol sequences ([Bibr bib1.bibx44]), applied simultaneously on two radio frequency channels.

The PulsePol sequence was originally developed in the context of electron–nucleus polarisation transfer ([Bibr bib1.bibx44]). As discussed in [Bibr bib1.bibx42], PulsePol may be interpreted as a “riffled” implementation of an R sequence, using the nomenclature of symmetry-based recoupling in solid-state NMR ([Bibr bib1.bibx10]). In the case of PulsePol, the R element is a composite $90_{y}180_{x}90_{y}$ pulse, with “windows” inserted between the pulses. Furthermore, in the current implementation, the central $180_{x}$ pulse of each R element is itself substituted by a BB1 composite pulse ([Bibr bib1.bibx48]). That substitution was previously shown to increase the robustness of the pulse sequence with respect to deviations in the radio frequency amplitudes and resonance offsets ([Bibr bib1.bibx42]). The total R-element duration, including all pulses and windows, is denoted using $\tau_{R}$ here (see Fig. [Fig Ch1.F4]).

For the experiments described here, the DualPol sequences used an R-element duration equal to $\tau_{R}$ = 70 ms , with pulse durations given by $\tau_{90}$ = 62.5 µs for the 90° pulses and $\tau_{\text{BB1}}$ = 10 × $\tau_{90}$ = 625 µs for the BB1 composite 180° pulses.

The ${}^{103}\mathrm{Rh}$ and methine ${}^{1}H$ nuclei of ${}^{103}\mathrm{Rh}(\mathrm{acac}{)}_{3}$ form an $I_{3}\;S$ spin system, where the ${}^{103}\mathrm{Rh}$ nucleus is the S spin and the magnetically equivalent ${}^{1}H$ nuclei are the I spins.

The DualPol spin dynamics are identical to those for Hartmann–Hahn J cross-polarisation ([Bibr bib1.bibx12]). The DualPol average Hamiltonian has the following form:

1 $${\bar{H}}^{\left(1\right)}=\kappa \times 2\pi J_{\text{IS}}(I_{x}S_{x}+I_{y}S_{y}),$$

where the scaling factor is given by κ=1/2 in the limit of short, ideal, radio frequency pulses. In the absence of relaxation and pulse imperfections, a DualPol sequence with a scaling factor κ=1/2 , applied to an $I_{3}\;S$ spin system should give rise to the following enhancement of the S-spin magnetisation, relative to its thermal-equilibrium value:

2 $$\begin{array}{rl} \epsilon_{\text{DualPol}}(T)= & \;\frac{\gamma_{I}}{4\gamma_{S}}\left\{2{\mathrm{sin}}^{2}\left(\frac{1}{2}J_{\text{IS}}T\right)\right.\\ & \left.+{\mathrm{sin}}^{2}\left(\pi J_{\text{IS}}T\right)+2{\mathrm{sin}}^{2}\left(\frac{1}{2}\sqrt{3}\pi J_{\text{IS}}T\right)\right\}. \end{array}$$

Here, T is the overall duration of the DualPol sequence. As the magnetogyric ratios of ${}^{103}\mathrm{Rh}$ and ${}^{1}H$ have opposite signs, the function $\epsilon_{\text{DualPol}}(T)$ is negative for all values of T . The blue curve in Fig. [Fig Ch1.F5] shows a plot of $|\epsilon_{\text{DualPol}}|$ against T , for a J coupling of $|J_{\text{IS}}|$ ≃ 0.39 Hz . The maximum value of $|\epsilon_{\text{DualPol}}|$ is given in the absence of relaxation by

3 $$\left|\epsilon_{\text{DualPol}}^{\text{max}}\right|=|17\gamma_{I}/16\gamma_{S}|≃33.56$$

for the case of I = ${}^{1}H$ and S = ${}^{103}\mathrm{Rh}$ . As Eq. ([Disp-formula Ch1.E2]) is quasi-periodic ([Bibr bib1.bibx12]), the value of T which maximises $|\epsilon_{\text{DualPol}}|$ is indeterminate. The first maximum may be found numerically and occurs at the duration $T_{1\text{st max}}$ ≃ $0.6098J_{\text{IS}}^{-1}$ , at which point the theoretical enhancement is given by $|\epsilon_{\text{DualPol}}|$ ≃ $1.052|\gamma_{I}/\gamma_{S}|$ ≃ 33.23 for the case of I = ${}^{1}H$ and S = ${}^{103}\mathrm{Rh}$ . Hence, for the estimated ${}^{1}H$ – ${}^{103}\mathrm{Rh}$ J coupling of $|J_{\text{IS}}|≃$ 0.39 Hz , assuming κ=1/2 , the ${}^{103}\mathrm{Rh}$ signal enhancement is expected to reach its first maximum at a DualPol duration of $T_{1\text{st max}}$ ≃ 1.563 s , in the absence of relaxation.

**Figure 5 Ch1.F5:**
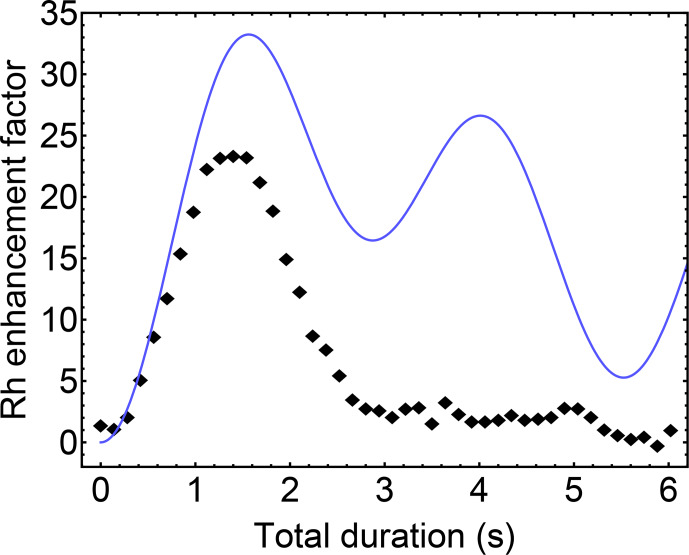
${}^{103}\mathrm{Rh}$ signal enhancement factor for ${}^{103}\mathrm{Rh}(\mathrm{acac}{)}_{3}$ as a function of DualPol sequence duration T , normalised against thermal-equilibrium ${}^{103}\mathrm{Rh}$ polarisation. Black diamonds: experimental data points; solid blue line: the theoretical enhancement factor $|\epsilon_{\text{DualPol}}(T)|$ for an $I_{3}\;S$ spin system in the absence of relaxation, as given by Eq. ([Disp-formula Ch1.E2]) for $|J_{\text{IS}}|$ = 0.39 Hz .

In the experiments described here, the optimum duration of the DualPol sequence was found for a repetition number of n=11 . For an R-element duration of $\tau_{R}$ = 70 ms , this corresponds to a total DualPol sequence duration of T = 1.54 s , which is in good agreement with the theoretical value.

The DualPol-enhanced ${}^{103}\mathrm{Rh}$ spectrum is shown in Fig. [Fig Ch1.F3]b and displays an experimental signal enhancement of ∼ 23 over the directly excited ${}^{103}\mathrm{Rh}$ spectrum in Fig. [Fig Ch1.F3]a.

Figure [Fig Ch1.F5] shows the experimental ${}^{103}\mathrm{Rh}$ signal enhancement factor as a function of the DualPol sequence duration T . Although the maximum of the experimental enhancement occurs at a similar position to the maximum of the theoretical curve, there is clearly a strong damping of the enhancement with respect to the duration T , leading to a loss of intensity at the theoretical maximum. This damping may be associated with transverse relaxation of the ${}^{1}H$ and ${}^{103}\mathrm{Rh}$ transverse magnetisation during the polarisation transfer process.

#### 3.2.3 ${}^{1}H$ – ${}^{103}\mathrm{Rh}$ polarisation transfer by refocused INEPT

Polarisation transfer from ${}^{1}H$ to ${}^{103}\mathrm{Rh}$ may also be conducted by the standard refocused-INEPT pulse sequence [Bibr bib1.bibx7]. In this case, the theoretical enhancement of the S-spin magnetisation, due to transfer from the I spins, is given for the $I_{3}\;S$ case, in the absence of relaxation and other imperfections, by [Bibr bib1.bibx7]:

4 $$\begin{array}{rl} \epsilon_{\text{RI}}(\tau_{1},\tau_{2})= & \;\frac{3\gamma_{I}}{4\gamma_{S}}\mathrm{sin}(\pi J_{\text{IS}}\tau_{1})\\ & \times \left(\mathrm{sin}(\pi J_{\text{IS}}\tau_{2})+\mathrm{sin}(3\pi J_{\text{IS}}\tau_{2})\right), \end{array}$$

where $\tau_{1}$ and $\tau_{2}$ refer to the total echo durations including two inter-pulse intervals, as shown in Fig. 1 of [Bibr bib1.bibx7]. The maximum of this function is found at $\tau_{1}=(2J_{\text{IS}}{)}^{-1}$ and $\tau_{2}=\text{arcsin}(3^{-1/2})/(\pi J_{\text{IS}})$ , giving an enhancement of $|\epsilon_{\text{RI}}|=|2\gamma_{I}/\sqrt{3}\gamma_{S}|$ ([Bibr bib1.bibx17]). Therefore, the maximum theoretical enhancement by refocused INEPT is

5 $$\left|\epsilon_{\text{RI}}^{\text{max}}\right|=|2\gamma_{I}/\sqrt{3}\gamma_{S}|≃1.155|\gamma_{I}/\gamma_{S}|≃36.48$$

for the case of I = ${}^{1}H$ and S = ${}^{103}\mathrm{Rh}$ . Hence, in the absence of relaxation, refocused INEPT can give a slightly greater enhancement than DualPol in an $I_{3}\;S$ system. However, the maximum enhancements by both DualPol and INEPT are less than the theoretical bound on the enhancement of S-spin magnetisation by polarisation transfer from the I spins in a permutation-symmetric $I_{3}\;S$ spin system, which is equal to $|3\gamma_{I}/2\gamma_{S}|$ ([Bibr bib1.bibx37]).

The theoretical advantage of INEPT over DualPol is not realised in practice for the case of ${}^{103}\mathrm{Rh}(\mathrm{acac}{)}_{3}$ . The maximum enhancement by refocused INEPT was realised for durations of $\tau_{1}$ = 920 and $\tau_{2}$ = 500 ms , which yielded a ${}^{103}\mathrm{Rh}$ enhancement factor of ∼ 17 over thermal polarisation, i.e. less than the maximum DualPol enhancement, which was ∼ 23. The experimentally optimised interval $\tau_{1}$ is significantly shorter than the optimum theoretical value in the absence of relaxation, which is $\tau_{1}^{\text{theor}}$ = 1.28 s , assuming a ${}^{103}\mathrm{Rh}$ – ${}^{1}H$ spin–spin coupling of $J_{\text{HRh}}$ = 0.39 Hz . The optimum value of $\tau_{2}$ , on the other hand, is very similar to the theoretical value, which is $\tau_{2}^{\text{theor}}$ = 503 ms .

The ${}^{1}H$ -enhanced ${}^{103}\mathrm{Rh}$ spectrum, produced by an optimised refocused-INEPT sequence, is shown in Fig. [Fig Ch1.F3]c. It shows a significantly lower enhancement than the DualPol result of Fig. [Fig Ch1.F3]b, despite the fact that the theoretical enhancement by refocused INEPT is higher than that of DualPol in the absence of relaxation (see Eqs. [Disp-formula Ch1.E3] and [Disp-formula Ch1.E5]). The loss of amplitude relative to the theoretical values may be attributed to transverse ${}^{1}H$ relaxation during the polarisation transfer process. It is known that Hartmann–Hahn-style cross-polarisation sequences such as DualPol can outperform INEPT in the presence of transverse relaxation ([Bibr bib1.bibx31]).

**Figure 6 Ch1.F6:**
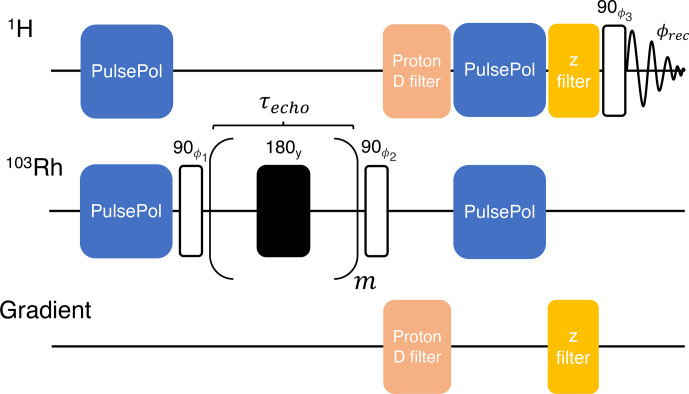
Sequence used for the indirect measurement of rhodium $T_{2}$ through ${}^{1}H$ detection. The phase cycles are given by the following: $\phi_{1}=[x,x,-x,-x]$ , $\phi_{2}=[-x,x,-x,x]$ , $\phi_{3}=[x,x,x,x,y,y,y,y,-x,-x,-x,-x,-y,-y,-y,-y]$ and the receiver $\phi_{\text{rec}}=[x,-x,-x,x,y,-y,-y,y,-x,x,x,-x,-y,y,y,-y]$ . The echo interval $\tau_{\text{echo}}$ was 45 ms . The black rectangle indicates a symmetrised BB1 composite 180° pulse ([Bibr bib1.bibx48]). An MLEV-64 supercycle was applied to the phases of the 180° pulses ([Bibr bib1.bibx32]). The ${}^{1}H$ “D-filter” and “z-filter” modules are described in Figs. 3 and 4 of [Bibr bib1.bibx25], respectively.

## 4 ${}^{1}H$ -detected ${}^{103}\mathrm{Rh}$ $T_{2}$

The ${}^{103}\mathrm{Rh}$ $T_{2}$ relaxation time constant for ${}^{103}\mathrm{Rh}(\mathrm{acac}{)}_{3}$ in ${\mathrm{CDCl}}_{3}$ was measured via the methine ${}^{1}H$ signals using a variant of a previously described indirectly detected $T_{2}$ DualPol pulse sequence ([Bibr bib1.bibx25]), which is shown in Fig. [Fig Ch1.F6]. The pulse sequence starts with a DualPol sequence of duration T = 1.54 s to transfer thermal-equilibrium longitudinal ${}^{1}H$ magnetisation to ${}^{103}\mathrm{Rh}$ . The ${}^{103}\mathrm{Rh}$ longitudinal magnetisation is converted into ${}^{103}\mathrm{Rh}$ transverse magnetisation by a 90° pulse on the ${}^{103}\mathrm{Rh}$ channel. The ${}^{103}\mathrm{Rh}$ transverse magnetisation evolves under a Carr–Purcell–Meiboom–Gill (CPMG) train of m spin echoes, each with an echo duration $\tau_{\text{echo}}$ = 45 ms . The Carr–Purcell sequence suppresses the confounding effects of translational diffusion and mixing with antiphase spin operators ([Bibr bib1.bibx40]). The ${}^{103}\mathrm{Rh}$ transverse magnetisation is converted to ${}^{103}\mathrm{Rh}$ longitudinal magnetisation by a second 90° ${}^{103}\mathrm{Rh}$ pulse. A ${}^{1}H$ “D-filter” module is applied to destroy any residual ${}^{1}H$ magnetisation, before another DualPol sequence of duration T = 1.54 s transfers the ${}^{103}\mathrm{Rh}$ longitudinal magnetisation to ${}^{1}H$ longitudinal magnetisation. A ${}^{1}H$ “z-filter” module is applied to destroy any other ${}^{1}H$ magnetisation components, followed by a 90° ${}^{1}H$ pulse which excites ${}^{1}H$ transverse magnetisation whose precession induces a ${}^{1}H$ NMR signal which is detected in the following interval. The ${}^{1}H$ D-filter and z-filter modules are described in Figs. 3 and 4 of [Bibr bib1.bibx25], respectively.

Repetition of the experiment with increasing values of m leads to the ${}^{103}\mathrm{Rh}$ $T_{2}$ decay curve shown in Fig. [Fig Ch1.F7]. This fits well to a single exponential decay with the time constant $T_{2}{(}^{103}\text{Rh})$ = 18.36 ± 0.92 s .

**Figure 7 Ch1.F7:**
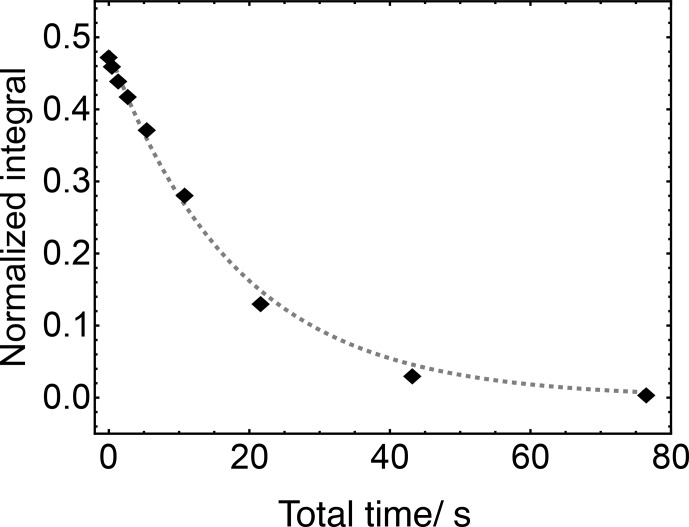
Decay curve for the ${}^{103}\mathrm{Rh}$ transverse magnetisation of ${}^{103}\mathrm{Rh}(\mathrm{acac}{)}_{3}$ in solution at a field of 9.4 T , measured by the indirectly detected multiple spin-echo scheme in Fig. [Fig Ch1.F6]. The experimental duration was 15 min .

**Figure 8 Ch1.F8:**
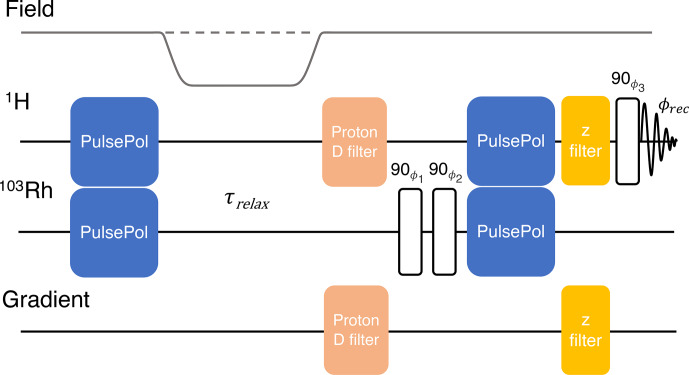
Sequence used for the indirect measurement of the rhodium $T_{1}$ through the ${}^{1}H$ NMR signal. Phase cycles are given by the following: $\phi_{1}=[x,x,-x,-x]$ , $\phi_{2}=[-x,x,-x,x]$ , $\phi_{3}=[x,x,x,x,y,y,y,y,-x,-x,-x,-x,-y,-y,-y,-y]$ and the receiver $\phi_{rec}=[x,-x,-x,x,y,-y,-y,y,-x,x,x,-x,-y,y,y,-y]$ . The optional shuttling of the sample to low field, and back again, during the interval $\tau_{\text{relax}}$ is indicated. The ${}^{1}H$ “D-filter” and “z-filter” modules are described in Figs. 3 and 4 of [Bibr bib1.bibx25], respectively.

## 5 ${}^{1}H$ -detected ${}^{103}\mathrm{Rh}$ $T_{1}$

The ${}^{103}\mathrm{Rh}$ $T_{1}$ relaxation time constant for ${}^{103}\mathrm{Rh}(\mathrm{acac}{)}_{3}$ in ${\mathrm{CDCl}}_{3}$ was measured indirectly using the methine ${}^{1}H$ signals, by means of the pulse sequence shown in Fig. [Fig Ch1.F8] ([Bibr bib1.bibx25]). The pulse sequence starts with a DualPol sequence of duration T = 1.54 s to transfer thermal-equilibrium longitudinal ${}^{1}H$ magnetisation to ${}^{103}\mathrm{Rh}$ . For variable-field experiments, the sample is shuttled out of the high-field magnet into a low-field region. The nuclear magnetisation is allowed to relax for an interval $\tau_{\text{relax}}$ . If necessary, the sample is shuttled back into high field, and residual ${}^{1}H$ magnetisation is destroyed by a ${}^{1}H$ D-filter module. A pair of phase-cycled 90° ${}^{103}\mathrm{Rh}$ pulses are applied to select for ${}^{103}\mathrm{Rh}$ z-magnetisation before a second DualPol sequence of duration T = 1.54 s transfers the partially relaxed longitudinal ${}^{103}\mathrm{Rh}$ magnetisation to ${}^{1}H$ magnetisation. A ${}^{1}H$ z-filter module is applied to destroy any other ${}^{1}H$ magnetisation components, followed by a 90° ${}^{1}H$ pulse which excites ${}^{1}H$ transverse magnetisation whose precession induces a ${}^{1}H$ NMR signal that is detected in the following interval. The ${}^{1}H$ D-filter and z-filter modules are described in Figs. 3 and 4 of [Bibr bib1.bibx25], respectively. During $\tau_{\text{relax}}$ , ${}^{1}H$ -enhanced ${}^{103}\mathrm{Rh}$ magnetisation decays toward thermal ${}^{103}\mathrm{Rh}$ magnetisation, which is very small; hence, at large values of $\tau_{\text{relax}}$ , resulting ${}^{103}\mathrm{Rh}$ -derived ${}^{1}H$ signals are very weak and close to zero, even for measurements performed at higher magnetic field strengths.

**Figure 9 Ch1.F9:**
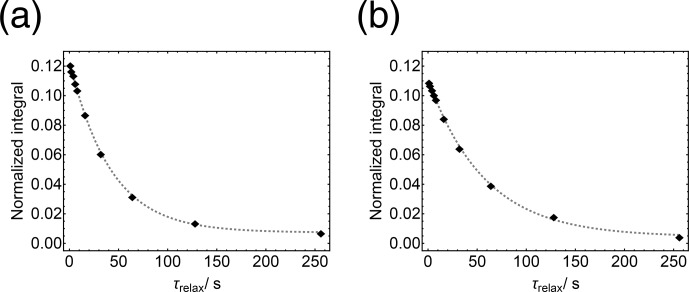
Trajectories of longitudinal ${}^{103}\mathrm{Rh}$ magnetisation for ${}^{103}\mathrm{Rh}(\mathrm{acac}{)}_{3}$ in solution at a temperature of 295 K , measured indirectly through the methine ${}^{1}H$ signals, using the pulse sequence in Fig. [Fig Ch1.F8]. **(a)** Filled symbols: ${}^{1}H$ signal amplitudes at a magnetic field of 9.4 T . The data were acquired in ∼ 2 h . The integrals are normalised against the ${}^{1}H$ spectrum obtained by a single ${}^{1}H$ 90° pulse applied to a system in thermal equilibrium at 9.4 T and at 295 K . Dotted line: fitted exponential decay with time constant $T_{1}({}^{103}\mathrm{Rh})$ = 41.8 ± 0.9 s . Panel **(b)** is the same as panel **(a)** but the sample is shuttled to a field of 10 mT during the relaxation delay $\tau_{\text{relax}}$ . Dotted line: fitted exponential decay with time constant $T_{1}({}^{103}\mathrm{Rh})$ = 57.8 ± 1.7 s .

The trajectory of indirectly detected ${}^{103}\mathrm{Rh}$ z-magnetisation in a field of 9.4 T and at a temperature of 295 K is shown in Fig. [Fig Ch1.F9]a. The trajectory fits well to a single exponential decay with time constant $T_{1}({}^{103}\mathrm{Rh})$ = 41.8 ± 0.9 s . A trajectory in the low magnetic field of 10 mT and at a temperature of 295 K is shown in Fig. [Fig Ch1.F9]b. This was produced by shuttling the sample to a low magnetic field, and back again, during the interval $\tau_{\text{relax}}$ . The relaxation process is somewhat slower in a low magnetic field, with a time constant of $T_{1}({}^{103}\mathrm{Rh})$ = 57.8 ± 1.7 s .

**Figure 10 Ch1.F10:**
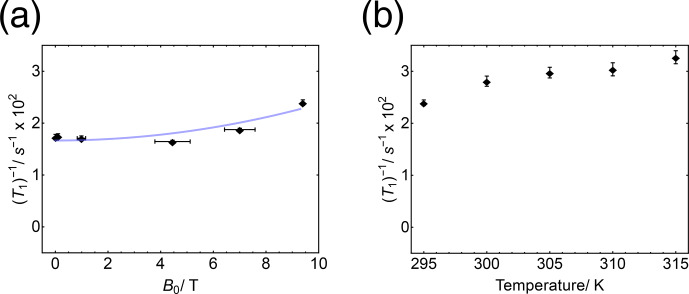
**(a)** ${}^{103}\mathrm{Rh}$ relaxation rate constant $T_{1}^{-1}$ for ${}^{103}\mathrm{Rh}(\mathrm{acac}{)}_{3}$ in solution, as a function of magnetic field strength at a temperature of 295 K . The blue line shows the best-fit quadratic function $T_{1}^{-1}(B)=T_{1}^{-1}(0)+aB^{2}$ , where $T_{1}^{-1}(0)$ = (167 ± 7) × 10 ${}^{-4}$ $s^{-1}$ and a = (7 ± 2) × 10 ${}^{-5}$ $s^{-1}\;T^{-2}$ . **(b)** ${}^{103}\mathrm{Rh}$ relaxation rate constant $T_{1}^{-1}$ for ${}^{103}\mathrm{Rh}(\mathrm{acac}{)}_{3}$ in solution, as a function of temperature at a magnetic field strength of 9.4 T .

The observed field dependence of the ${}^{103}\mathrm{Rh}$ relaxation rate constant $T_{1}^{-1}$ is shown in Fig. [Fig Ch1.F10]a. The magnetic field dependence of $T_{1}^{-1}$ is quite weak in this range of fields. The relaxation rate constant increases slightly with increasing magnetic field at the high-field end, suggestive of a weak relaxation contribution from the ${}^{103}\mathrm{Rh}$ chemical shift anisotropy. The blue curve in Fig. [Fig Ch1.F10]a shows the best-fit quadratic function $T_{1}^{-1}(B)=T_{1}^{-1}(0)+aB^{2}$ , where $T_{1}^{-1}(0)$ = (167 ± 7) × 10 ${}^{-4}$ $s^{-1}$ and a = (7 ± 2) × 10 ${}^{-5}$ $s^{-1}\;T^{-2}$ .

The observed temperature dependence of the ${}^{103}\mathrm{Rh}$ relaxation rate constant $T_{1}^{-1}$ is shown for a field of B ≃ 9.4 T in Fig. [Fig Ch1.F10]b. The rhodium $T_{1}^{-1}$ increases monotonically with increasing temperature over the relevant temperature range. At 315 K , relaxation occurs with a time constant of $T_{1}({}^{103}\mathrm{Rh})$ = 30.6 ± 1.1 s .

A positive dependence of the ${}^{103}\mathrm{Rh}$ $T_{1}^{-1}$ on temperature was reported previously for ${}^{103}\mathrm{Rh}(\mathrm{acac}{)}_{3}$ in solution ([Bibr bib1.bibx3]).

## 6 Discussion

The temperature dependence of the ${}^{103}\mathrm{Rh}$ $T_{1}^{-1}$ , as shown in Fig. [Fig Ch1.F10]b, indicates a dominant spin–rotation relaxation mechanism. For small molecules with a short rotational correlation time relative to the nuclear Larmor period, spin–rotation is the only mechanism that leads to a positive correlation of $T_{1}^{-1}$ with temperature ([Bibr bib1.bibx30]). This is because the amplitudes of the local magnetic fields generated by the spin–rotation interaction are proportional to the root-mean-square rotational angular momentum of the participating molecules – a quantity that is linked to the mean rotational kinetic energy of the molecules, which increases linearly with temperature. For other mechanisms, the decrease in the rotational correlation time $\tau_{c}$ with increasing temperature leads to a decrease in the relaxation rate with increasing temperature, in the fast-motion limit.

The field dependence of the ${}^{103}\mathrm{Rh}$ $T_{1}^{-1}$ , as shown in Fig. [Fig Ch1.F10]a, displays a modest increase in relaxation rate with increasing magnetic field at high field, which suggests an additional contribution from the rotational modulation of the ${}^{103}\mathrm{Rh}$ CSA tensor. A finite ${}^{103}\mathrm{Rh}$ CSA tensor is allowed with respect to symmetry under the $D_{3}$ point group of ${}^{103}\mathrm{Rh}(\mathrm{acac}{)}_{3}$ ([Bibr bib1.bibx6]). Indeed, solid-state NMR data indicate a ${}^{103}\mathrm{Rh}$ shielding anisotropy of Δσ ≃ 460 ppm , with relativistic quantum chemistry calculations in reasonable agreement ([Bibr bib1.bibx29]). The magnitude of this CSA tensor is modest by ${}^{103}\mathrm{Rh}$ standards. For example, the ${}^{103}\mathrm{Rh}$ nuclei in Rh paddlewheel complexes have a shielding anisotropy of |Δσ| ∼ 9900 ppm ([Bibr bib1.bibx25]).

In summary, we have demonstrated the successful transfer of polarisation between the central ${}^{103}\mathrm{Rh}$ nucleus and the three methine ${}^{1}H$ nuclei in ${}^{103}\mathrm{Rh}(\mathrm{acac}{)}_{3}$ , through the very small four-bond ${}^{1}H$ – ${}^{103}\mathrm{Rh}$ couplings. The polarisation transfer is more efficient for DualPol than for refocused INEPT, even though the theoretical efficiency of DualPol is slightly less than that of refocused INEPT, for the relevant $I_{3}\;S$ spin system. We have successfully exploited ${}^{1}H$ – ${}^{103}\mathrm{Rh}$ polarisation transfer to study the longitudinal and transverse relaxation of ${}^{103}\mathrm{Rh}$ for ${}^{103}\mathrm{Rh}(\mathrm{acac}{)}_{3}$ in solution. The ${}^{103}\mathrm{Rh}$ $T_{1}$ relaxation is dominated by the spin–rotation mechanism, with a significant additional contribution from the ${}^{103}\mathrm{Rh}$ CSA at a high magnetic field.

## Data Availability

The dataset can be accessed at 10.5258/SOTON/D3209 ([Bibr bib1.bibx14]).

## Code availability

The software code for the graphics shown in this paper is available from the authors on reasonable request.
